# Implication of Tumor Microenvironment in Chemoresistance: Tumor-Associated Stromal Cells Protect Tumor Cells from Cell Death

**DOI:** 10.3390/ijms13089545

**Published:** 2012-07-30

**Authors:** Magali Castells, Benoît Thibault, Jean-Pierre Delord, Bettina Couderc

**Affiliations:** EA4553, Institut Claudius Regaud, F-31062 Toulouse, France and University of Toulouse III, Toulouse F-31052, France; E-Mails: castells.magali@claudiusregaud.fr (M.C.); thibault.benoit@claudiusregaud.fr (B.T.); delord.jean-pierre@claudiusregaud.fr (J.-P.D.)

**Keywords:** microenvironment, cancer, chemoresistance, apoptosis

## Abstract

Tumor development principally occurs following the accumulation of genetic and epigenetic alterations in tumor cells. These changes pave the way for the transformation of chemosensitive cells to chemoresistant ones by influencing the uptake, metabolism, or export of drugs at the cellular level. Numerous reports have revealed the complexity of tumors and their microenvironment with tumor cells located within a heterogeneous population of stromal cells. These stromal cells (fibroblasts, endothelial or mesothelial cells, adipocytes or adipose tissue-derived stromal cells, immune cells and bone marrow-derived stem cells) could be involved in the chemoresistance that is acquired by tumor cells via several mechanisms: (i) cell–cell and cell–matrix interactions influencing the cancer cell sensitivity to apoptosis; (ii) local release of soluble factors promoting survival and tumor growth (crosstalk between stromal and tumor cells); (iii) direct cell-cell interactions with tumor cells (crosstalk or oncologic trogocytosis); (iv) generation of specific niches within the tumor microenvironment that facilitate the acquisition of drug resistance; or (v) conversion of the cancer cells to cancer-initiating cells or cancer stem cells. This review will focus on the implication of each member of the heterogeneous population of stromal cells in conferring resistance to cytotoxins and physiological mediators of cell death.

## 1. Introduction

Most current treatments for peritoneal adenocarcinoma consist of chemotherapy associated with surgery. For example, the standard treatment of ovarian adenocarcinoma is cytoreductive surgery pre and post chemotherapy. Most patients are chemosensitive and cancer free immediately after the treatment. However, depending on the quality of the surgery, 50% to 70% of patients will relapse within one year. When such relapse occurs, in most cases the adenocarcinoma cells have acquired a chemoresistant phenotype. This chemoresistance can be associated with genetic alterations within the cancer cells but recent studies have proposed that it could also be associated with the tumor microenvironment [1]. Indeed, this microenvironment has become recognized as a major factor influencing the growth of cancer and impacting the outcome of therapy. While the niche cells are not malignant per se, their role in supporting cancer growth is so vital for the survival of the tumor that they have become an attractive target for chemotherapeutic agents [2]. Meads *et al*. have shown that environment-mediated drug resistance is rapidly induced by signaling events from the tumor microenvironment and is likely to be reversible because removal of the microenvironment restores the drug sensitivity [1,3].

The microenvironment (stroma) is made up of endothelial cells, carcinoma-associated fibroblasts (CAFs), adipocytes, mesenchymal cells, mesenchymal stem cells (MSCs; bone marrow derived, BM-MSCs, or carcinoma associated, CA-MSCs), and cells from the immune and inflammatory systems (tumor-associated macrophages, TAM, regulatory T cells, *etc*.). The stromal cells crosstalk not only with tumor cells but also with each other [4]. Microenvironment cells could potentially induce chemoresistance acquisition in tumor cells by: (i) cell–cell and cell–matrix interactions influencing the cancer cell sensitivity to apoptosis and thereby affecting drug resistance; (ii) local release of soluble factors such as interleukin-6 (IL-6) that promote survival and tumor growth (crosstalk between stromal cells and tumor cells through paracrine factors secreted by the two cell populations) [1,5,6]; (iii) direct cell-cell interactions with tumor cells (crosstalk or oncologic trogocytosis) [7]; iv) the generation of specific niches within the tumor microenvironment comprising subpopulations of tumor cells that may afford a survival advantage following initial drug exposure and/or enhance hypoxia leading to the up-regulation of growth factors such as platelet-derived growth factor B (PDGF-B), transforming growth factor β (TGF-β), insulin-like growth factor 2 (IGF-2), epidermal growth factor (EGF) by the stromal cells and allowing a paradoxal increase in cell chemoresistance [8,9]; (v) the conversion of the cancer cells into cancer-initiating cells or cancer stem cells [10].

## 2. Cell Adhesion and Chemoresistance

There are “dynamic and reciprocal” exchanges of information between tumor cells and their surrounding. A permissive microenvironment could affect the sensitivity of tumor cells to drug treatment. The composition and organization of the ECM (extracellular matrix) and stromal components contribute to marked gradients in drug concentration, increasing interstitial fluid pressure and metabolic changes, all of which can strongly enhance the resistance of tumor cells to drug agents [11]. The importance of the microenvironment and the structural organization of cells in a 3D context have been recognized for decades. Indeed, in 1979, Sutherland *et al*. showed that the resistance of mammary tumor cells to adriamycin was higher when the cells were organized spheroidally compared to in a monolayer [12]. This increased chemoresistance is not due to reduced drug internalization but to the interaction between cells and the extracellular matrix. Over the past decade, a number of studies have demonstrated the increased survival of several tumor models induced by the adhesion of cancer cells, via CAMs and integrins, to extracellular matrix components like fibronectin or laminin [11]. As an example, L1-cell adhesion molecule (L1-CAM) can prevent cisplatin-induced apoptosis in the ovarian carcinoma cell line OVMz [13]. Concordantly, the association of an L1-CAM blocking antibody with cytostatic drugs reduces tumor growth *in vivo* in pancreatic and ovarian carcinoma models in relation to an increased number of apoptotic cells, shown by an increased expression of procaspase-8, and to caspase-3 activation [14]. Erbele *et al*. showed that a cisplatin treatment can trigger the proliferation of oral carcinoma cells adherent to a carcinoma matrix through integrin β1 and NF-κB dependent pathways [15]. In the same way, integrin binding to ECM and stromal cells can also control cell cycle progression in both haematological and epithelial malignancies. Correai *et al*. described in their review works made by Hazlehurst *et al*. who reported that G1 arrest of myeloma cells induced by β1 integrin adhesion to fibronectin correlates with upregulated levels of cells regulator p27, and enhanced resistance to etoposide [11,16]. At last aside from determining cell and tissue architecture, the way cell surface adhesion molecules perceive ECM also affect nuclear structure and chromatin organization. This chromatin reorganization can affect resistance to drugs which bind or disrupt DNA [11].

**Figure 1:**
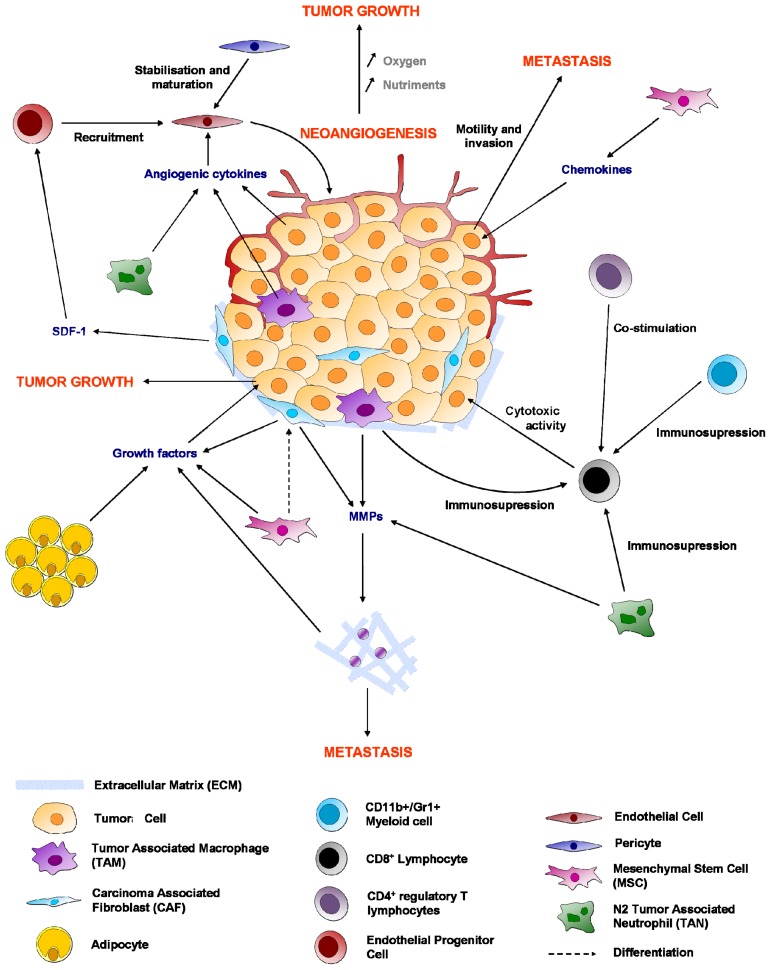
Non-exhaustive interactions between cancer and its microenvironment. The recruitment of endothelial cells and pericytes by the tumor leads to the formation of new vessels and an increased supply of oxygen and nutrients. Within the tumor site, immune cells usually acquire a tumor-associated immunosuppressive phenotype, except for cytotoxic CD8^+^ lymphocytes which kill cancer cells. Pro-tumoral effects of adipocytes, carcinoma associated fibroblasts (CAFs) or mesenchymal stem cells (MSCs) include the secretion of growth factors enhancing tumor growth and the secretion of matrix metalloproteases (MMPs) which degrade the extracellular matrix and potentialize metastasis.

Together, these data show that a “malignant” tissue architectural phenotype is responsible for increased survival of tumors against chemotherapies (Figure 1).

## 3. Endothelial Cells

Blood vessels are an essential part of the tumor since they transport the nutrients and oxygen required for tumor survival and growth. Tumor vascularization, a process enabled by the “angiogenic switch”, induces a decrease in tumor cell apoptosis [17]. The angiogenesis mechanism is thus also responsible for tumor chemoresistance by promoting resistance to apoptosis.

### 3.1. Growth Factors Involved in Angiogenesis Are Pro Survival

Vascular endothelial growth factor (VEGF) is the most potent angiogenesis inducer. It stimulates endothelial cell proliferation, migration, differentiation and also vessel branching (reviewed recently in [18]). During the angiogenesis process, VEGF is secreted by several cells including endothelial cells and tumor cells [19].

Some cancer cells express one of the two VEGF receptors, VEGFR-1 or VEGFR-2 (Flk/KDR), and can be stimulated by VEGF signaling [20–22]. VEGF is not only a mitogen but also a survival factor [23] the overexpression of which can induce resistance to chemotherapeutic drugs in soft tissue sarcoma [24]. VEGFR-2 expression was correlated with chemoresistance in non-small-cell lung carcinoma [25] whereas the loss of VEGF expression in colorectal cancer cells caused an increase in apoptosis (spontaneous and chemotherapy induced) [26]. VEGF modifies the apoptotic signaling pathway in endothelial cells by inducing the expression of anti-apoptotic proteins including survivin and Bcl-2 [27–29], and the activation of the PI3K/Akt survival pathway [30]. Bcl-2 expression or phosphorylation can also be up-regulated by VEGF in VEGFR^+^ primary and/or immortalized cancer cells from different sources, leukemia [31,32] or in breast cancer [33]. In chronic lymphocytic leukemia B cells, VEGF interacts directly with STAT1 and STAT3 leading to the up-regulation of other anti-apoptotic proteins, Mcl-1 and XIAP, and protection from cell death [34]. Pericytes, specialized in the stabilization and maturation of vessels, can promote endothelial cell survival [35]. They act on endothelial cells by stimulating the expression of VEGF via NF-κB signaling. By secreting vitronectin, the pericytes induce the activation of endothelial cell integrin α_V_ leading to the increased expression of the anti-apoptotic protein Bcl-w [36]. Interestingly, such prosurvival signaling induced by VEGF can be converted into pro-cell death signals by TGFβ1, a regulator of tissue morphogenesis, during an apoptosis step required for angiogenesis [37].

Chemoresistance acquisition and angiogenesis are two linked processes. Indeed one study showed that chemoresistance acquisition by continuous treatment in neuroblastoma cells was related to transcriptome modifications in angiogenic genes and correlated with a positive influence on angiogenesis *in vitro* and *in vivo* [38]. In breast cancer cell lines, hypoxia-stimulated VEGF expression was increased by overexpression of Bcl-2 [39]. In contrast, down-regulation of anti-apoptotic proteins Bcl-2 or survivin by different approaches improved the sensitivity to treatment (radiotherapy or chemotherapy) and inhibited VEGF expression and angiogenesis in two different tumor xenograft models (prostate and colon) [40,41].

### 3.2. Endothelial Cell Protection against Apoptosis

In addition to the tumor cells, endothelial cells also need to be eradicated by any cancer treatment to minimize the risk of recurrence. These cells develop their own specific apoptosis resistance pathways depending on the model studied and death inducer. FGF-2, which is also an angiogenic factor, promotes apoptosis resistance in endothelial cells after radiation treatment [42] or growth factor deprivation [43]. It has the same prosurvival effects as VEGF, upregulating Bcl-2 and survivin expression [44] and activating the protein kinase Akt [45,46].

Both FGF-2 and VEGF protect endothelial cells from apoptosis after exposure to different chemotherapies, and have an additive effect due to activation of PI3K/Akt signaling pathway [26]. Cancer cells can act on endothelial cells and protect them from apoptosis after radiation through their secretion of VEGF and the subsequent activation of pro-survival gene expression [47]. Moreover, in different cancer models, tumor-associated endothelial cells have been shown to differ from those derived from normal organs or cells. For example, the tumor-associated endothelial cells showed increased chemoresistance compared to normal endothelial cells through the expression of PAX2 in renal carcinoma [48], the overexpression of survivin in a glioma model [49], and the activation of an NF-κB dependent pathway promoting Akt and VEGF expression and cell survival in hepatocellular carcinoma [50]. These molecular pathways are also associated with the stimulation of angiogenesis [48–50].

### 3.3. Treatments

Specific anti-angiogenic drugs have been developed over the last few years most of which target VEGF signaling (VEGF or VEGFR).

Anti-angiogenic therapies used as a single agent have been shown since the first preclinical studies to inhibit angiogenesis and diminish tumor growth [51] and also permit an increase in tumor cell apoptosis [52]. They are used in combination with conventional therapies for their ability to improve delivery [53] by reducing interstitial fluid pressure [54]. Moreover, the use of two angiogenic inhibitors together has shown very promising results in a glioma model where a VEGFR2 inhibitor alone did not permit blood vessel regression. A combination with an inhibitor of PDGFR-β overcame the survival mechanism by targeting pericytes, mediators of endothelial cell survival mechanisms, thus demonstrating that in blood vessels, resistant processes work cooperatively [55]. In clinical trials, anti-angiogenic therapies have been shown to improve the response to chemotherapy in different types of cancer [56,57] however when administered alone, they do not permit an improvement of long term benefit [58].

The paradox between anti-angiogenic therapy expectations and clinical observations is explained by the recent concept developing the idea that anti-angiogenic treatments provide the best results when they provoke “vessel normalization”. These drugs do not abrogate tumor angiogenesis but rather turn transiently anarchic tumor blood vessels, caused by overexpression of angiogenic factors, into normal ones. Not only does this allow a better delivery of chemotherapy and increase the sensitivity to radiotherapy but it also decreases tumor cell extravasation and migration in the blood circulation by reestablishing the endothelial cell barrier [55,59]. As with every therapy, resistance to an anti-angiogenic drug can occur mostly due to its mechanism of action. Indeed their aim is to suppress angiogenesis causing hypoxia [60].

### 3.4. From Hypoxia to Angiogenesis

To survive, tumor cells develop several adaptive mechanisms including metabolic shift (oxidative metabolism to glycolysis) and apoptosis resistance [61]. The microenvironment can positively influence tumor apoptosis resistance via the activation of resistance signaling pathways. Extreme conditions imposed by the microenvironment can also influence cancer cells by obliging them to modify their phenotype in order to overcome the hypoxia and survive. One of the major ways for tumors to survive in hypoxic conditions is to escape from the local area, thus explaining the high migration and invasion potential of these cells [60]. Hypoxia is drastic for cancer cells but leads to genetic instability and the selection of the most malignant cells with the highest metastatic abilities [62]. The key molecule acting during hypoxia is hypoxia-inducible transcription factor 1 (HIF-1) which transactivates hundreds of genes including angiogenic and autocrine growth factors and receptors, glycolytic enzymes and extracellular proteases [63]. Hypoxia particularly induces VEGF expression in tumor cells thereby activating anti-apoptotic pathways described in point 3.3. [64]. Hypoxia can also induce an inflammatory state through HIF1 and NF-κB activation leading to the secretion of chemokines and cytokines able to recruit inflammatory cells which also release VEGF [65].

Anti-angiogenic therapies, through the hypoxic response they cause in cancer cells, could be responsible for an enhancement of metastasis and invasion [66] and may bring about a more aggressive behavior. This point is controversial and still under investigation. Indeed, while some preclinical studies have shown that local invasiveness and metastasis are triggered by anti-VEGF treatments [67,68] others found no effect on metastasis [69,70].

## 4. Fibroblasts

### 4.1. Non Activated Fibroblasts

In the particular case of multiple myeloma (MM), a plasma cell cancer, the adhesion between MM cells and bone marrow fibroblasts leads to the secretion of IL-6 by the latter [71]. This pleiotropic cytokine has demonstrated the capacity to induce the resistance of MM cells to apoptotic stimuli and chemotherapeutic drugs via the Jak/STAT pathway and the expression of the anti-apoptotic protein Bcl-xL [72].

### 4.2. Myofibroblasts or Carcinoma-Associated Fibroblasts (CAFs)

Myofibroblasts or carcinoma-associated fibroblasts (CAFs) are the most abundant cell type in the tumor microenvironment. While their tumor-promoting effects are well known, their origin and a clearly characterized phenotype have not been well established. They can be distinguished from normal fibroblasts by their expression of certain markers like alpha smooth muscle actin (αSMA), fibroblast activation protein (FAP), tenascin-C or desmin [73].

Both cancer cells and CAFs are able to secrete prostaglandin E_2_ (generated by COX-2 activation) and sphingosine-1-phosphate (S1P), that can act in an autocrine or paracrine fashion to mediate cell survival and chemoresistance *via* PI3K-Akt/PKB pathway activation [74]. In cholangiocarcinoma, CAFs also secrete platelet growth factor BB (PDGF-BB) which protects the cholangiocarcinoma cells from TRAIL cytotoxicity thus implying involvement of the Hedgehog (Hh) pathway [75].

CAFs can regulate extracellular matrix composition by secreting periostin, a ligand of αvβ3 and αvβ5 integrins, which allows cancer cell adhesion and migration but also apoptosis resistance by PI3K-Akt/PKB activation in breast cancer models [76]. Thanks to their expression of the serine protease FAP, CAFs allow collagen I cleavage and thus extracellular matrix remodeling [77]. By interacting with collagen fibers in an integrin-dependent manner, these cells exert an increased tension between the fibers and ultimately increase the interstitial pressure which diminishes drug uptake and efficacy [78]. Concordantly, fibroblast-derived 3D matrix has been shown to promote resistance of the PANC-1 line (pancreatic cancer cells) to taxol [79].

Myofibroblasts have been shown to enhance chemoresistance in a pancreatic carcinoma model *via* an epigenetic inhibition of STAT1 and a reduced expression of caspases (8, 9, 7 and 3). They achieve this by inducing the expression of DNA methyltransferase 1 (DNMT1) and CpG DNA-hypermethylation [80].

Their abundance in the tumor stroma and various effects on tumor progression and apoptosis resistance has made CAFs a new target in anticancer therapy. The desire to target CAFs instead of normal fibroblasts drove the development of, among others, anti-tenascin-C or anti-FAP molecules. Results from a phase II trial of the anti-tenascin monoclonal antibody 81C6 followed by chemotherapy in malignant glioma were promising compared to the control group [81].

Stromal fibroblasts are genetically stable compared to tumor cells and constitute a reliable target for immunotherapy. Loeffler *et al*. developed a DNA vaccine which targets FAP by activating CD8^+^ T cells in order to specifically kill the CAFs and thereby decrease collagen I expression and enhance drug uptake. The combination of such an anti-FAP vaccine with doxorubicin brought about an inhibition of tumor growth and complete tumor rejection in half of the tested mice whereas there was no survival with doxorubicin treatment alone [82].

## 5. Mesenchymal Stem Cells (MSCs) or MSC-Like Cells (CA-MSCs)

**Figure 2:**
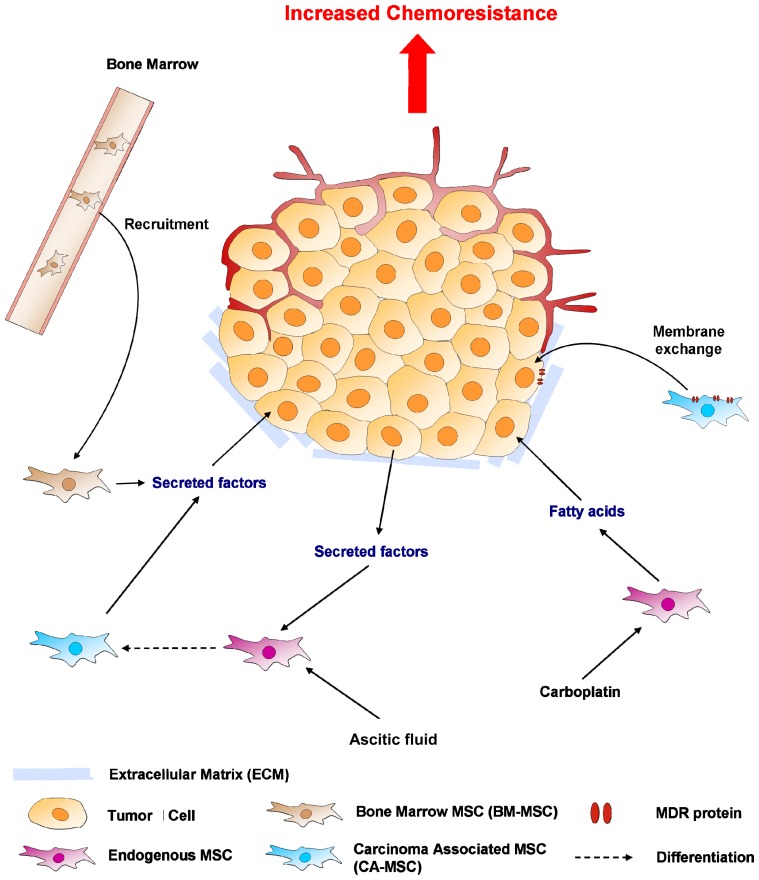
Chemoprotective effects of mesenchymal stem cells (MSCs) in ovarian cancer. In the ovarian stroma, MSCs present multiple phenotypes: they could be recruited from the bone marrow (BM-MSCs), established within the peritoneum (endogenous MSCs) or differentiated into a carcinoma-associated phenotype (CA-MSCs) via ovarian cancer cell secreted factors or molecules contained in the ascites. All these cells promote ovarian cancer cell resistance to carboplatin by several mechanisms including the secretion of unsaturated fatty acids (for endogenous MSCs activated by a carboplatin treatment) or that of factors with the exchange of MDR efflux pumps (for CA-MSCs).

MSCs are multipotent cells capable of differentiating into numerous cell types including adipocytes, osteoblasts, chondrocytes, fibroblasts, perivacular and vascular structures [3,83]. MSCs are recruited in large numbers to the stroma of developing tumors the growth of which induces a continuous production of paracrine and endocrine signals that mobilize the MSCs from the bone marrow (BM) [3]. In some peritoneal cancers such as in ovarian cancer, MSCs have been described as being located around the tumor cells and in the ascitic fluid. These MSCs can no longer be defined as multipotent as they are unable to differentiate in different cell lineages. They are defined as being carcinoma-associated (CA-MSCs) and present some characteristic markers of CAFs (expression of PDGFR, FAP, …) [84]. Such MSCs are found to stimulate tumor growth, enhance angiogenesis and promote metastasis formation through the release of a large spectrum of growth factors and cytokines [3,85,86]. Roodhart *et al*. [3], Xu *et al*. [87], Hao *et al*. [88], Jin *et al*. [89] and our groups [4,7] have recently shown that MSCs are also involved in the development of chemoresistance to multiple types of chemotherapies (Figures 1 and 2).

### 5.1. Cell-Cell Contact

MSCs could promote such chemoresistance by direct cell-cell contact. Firstly Xu *et al*. proposed that TGF-β1 produced by BM stromal cells promotes the survival and chemoresistance of leukemia cells via direct cell-to-cell interactions [87]. They showed that the blockade of TGF-β signaling by LY2109761, which effectively inhibited the pro-survival signaling, could enhance the efficacy of chemotherapy against myelo-monocytic leukemic cells in the BM microenvironment. Rafii *et al*. demonstrated the capacity of CA-MSCs (called Hospicells in their study) to confer chemoresistance to ovarian and breast cancer cells by direct cell-cell contact and the exchange of membrane patches and MDR proteins (oncologic trogocytosis) [5,7].

Jin *et al*. showed that MSC cocultured with KBM-5 leukemia cells protected the latter from imatinib-induced cell death. As these anti-apoptotic effects were abrogated by the CXCR4 antagonist AMD3465 or by the inhibitor of integrin-linked kinase QLT0267, they suggested that the upregulation of CXCR4 by imatinib promotes migration of chronic myelogenous leukemia (CML) cells to bone marrow stroma, causing G0-G1 cell cycle arrest and hence ensuring the survival of quiescent CML progenitor cells [89].

### 5.2. Soluble Factors Released Locally

Roodhart, Hao and Castells showed that MSCs could induce chemoresistance through the release of factors in the neighbourhood of tumors. Castells *et al*. showed that supernatants of CA-MSCs as well as BM-MSCs were able to induce development of chemoresistance by reducing apoptosis. They observed that CA-MSC secretions were able to confer carboplatin resistance to ovarian cancer cells by inhibiting the activation of effector caspases and apoptosis blockade. The activation of PI3K/Akt pathway signaling and phosphorylation of the downstream target Xiap underlined their implication in ovarian cancer chemoresistance.

The factors released in the tumor microenvironment have yet to be identified. Using activated MSCs, Roodhart *et al*. also showed that MSCs are potent mediators of resistance to chemotherapy and revealed targets to enhance chemotherapy efficacy in patients. They claimed that MSCs per se do not induce chemoresistance, rather endogenous MSCs become activated during treatment with platinum analogs and then secrete factors that protect tumor cells against a range of chemotherapeutic drugs. Through a metabolomic approach, they identified two distinct platinum-induced polyunsaturated fatty acids (PIFAs), 12-oxo-5,8,10-heptadecatrienoic acid (KHT) and hexadeca-4,7,10,13-tetraenoic acid (16:4(n-3)), that in minute quantities induce resistance to a broad spectrum of chemotherapeutic agents. Blocking central enzymes involved in the production of these PIFAs (cyclooxygenase-1 and thromboxane synthase) prevents such MSC-induced resistance [3]. Hao *et al*. demonstrated in myeloma that the secretion of IL-6, VEGF and cell-to-cell contact with microenvironment-derived stromal cells from patients with multiple myeloma (MM-BMSCs) significantly decreased the sensitivity of myeloma cells to bortezomib treatment. Mechanistically, they associated the chemoresistance to the suppression of miRNA-15a expression by the BM-MSCs [88].

### 5.3. Hypoxia

Pasquet *et al*. showed that CA-MSCs could induce the formation of hypoxic niches around tumors associated with a high expression of HIF1-α [84]. Benito *et al*. indicated that interactions between leukemia cells and the BM microenvironment promote leukemia cell survival and confer resistance to anti-leukemic drugs [90]. They correlated the hypoxic areas within the BM microenvironment with a survival advantage to hematopoietic cells and showed that hypoxia promotes chemoresistance in various ALL derived cell lines.

### 5.4. Conversion of Mesenchymal Cells to Cancer-Initiating Cells

In their study, Teng *et al*. have shown that DNA hypermethylation within a specific tumor suppressor gene is sufficient to fully transform a somatic mesenchymal stem cell. MSCs harboring targeted promoter methylations of HIC1/RassF1A displayed several features of cancer stem/initiating cells including loss of anchorage dependence, increased colony formation capability, drug resistance, and pluripotency. Moreover, the cells retained sensitivity to neuron- and osteocyte-induction and displayed both lineage-specific markers and stem cell markers in xenografts. Teng *et al*. proposed that under the influence of different environmental niches, these transformed stem cells could give rise to tissue-specific cancers [10].

## 6. Immune System

The immune system monitors and eliminates pathogens as well as developing tumors. However, tumor cells can escape from immunity and modify the phenotype of immune cells which become pro-tumor cells enhancing the tumor growth, angiogenesis or metastasis process [91]. Several mechanisms by which tumors escape from cell death induced by the immune system have already been described [92]. A few studies have been carried out on the effects of the immune system itself on the tumor resistance to chemotherapy and apoptosis.

Some recent studies reported the capacity of myeloid cells to mediate resistance to anti-angiogenic drugs. Shojaei *et al*. showed that CD11b^+^Gr1^+^ cells (which could be dendritic cells, monocytes or neutrophils) could be primed and recruited in tumors where they could mediate anti-VEGF treatment refractoriness. They also showed that combining anti-VEGF treatment and a monoclonal antibody targeting myeloid cells allowed a better growth inhibition than anti-VEGF alone in refractory tumors [93]. Targeting placental growth factor (PIGF), a pro-angiogenic cytokine, reduces tumor growth, angiogenesis and metastasis and enhances chemotherapy (gemcitabine or cyclophosphamide) and anti-VEGF therapy efficacy in melanoma or pancreatic tumor models. Fischer *et al*. showed that inhibition of the PIGF pathway was responsible for a reduced VEGFR-1^+^ macrophage recruitment leading to an improved response to anti-VEGF therapy [94].

### 6.1. Macrophages

Monocytes and macrophages derive from myeloid cells, which are located in the bone marrow. After maturation, monocytes circulate in the bloodstream and can migrate into tissues where they differentiate into macrophages. Depending on the environmental context and the tumor development stage, activated macrophages can be separated into two distinct phenotypes: M1 (classical activated), which inhibit tumor growth and M2 (alternative activated), which are pro-tumoral. While the exact definition is still controversial, it is clear that tumor-associated macrophages (TAMs) consistently present a highly immunosuppressive M2 profile [95].

Within developed tumors, there is a balance in favor of tumor progression and the chemoprotective effects of TAMs being closely associated with reduced cytotoxic CD8^+^ T-lymphocyte activity. Indeed, hypoxic TAMs suppress CD8^+^ T-lymphocytes activity by the activation of inducible Nitric Oxyde Synthase (iNOS) and arginase I (ArgI, liver-type) in a HIF1α-dependant manner. Moreover, the inhibition of TAM recruitment by a CSF1 neutralizing antibody in mammary tumors leads to a better chemosensitivity to paclitaxel, reduced tumor progression and metastasis that was associated with an increased survival of CD8^+^ T-cells. [96].

Shree *et al*. showed that cathepsin-expressing macrophages protect breast cancer cells from cell death induced by the following chemotherapeutic drugs: taxol, etoposide and doxorubicin. They highlighted the growing importance of combining anti-microenvironment drugs with classic chemotherapy. Indeed, the combination of anti-cathepsin with taxol treatment enhances the anti-tumor efficacy, the late-stage survival and decreases the metastatic burden compared to taxol alone in a breast cancer mouse model [97]. Mononuclear cells (monocyte-like cell line U937) prevent pancreatic cancer cells from camptothecin and genistein induced apoptosis *in vitro* by interleukin-1β-mediated expression of cycloxygenase-2 (COX-2) and the production of prostaglandins [98].

### 6.2. Cytotoxic CD8^+^ T Lymphocytes

**Figure 3:**
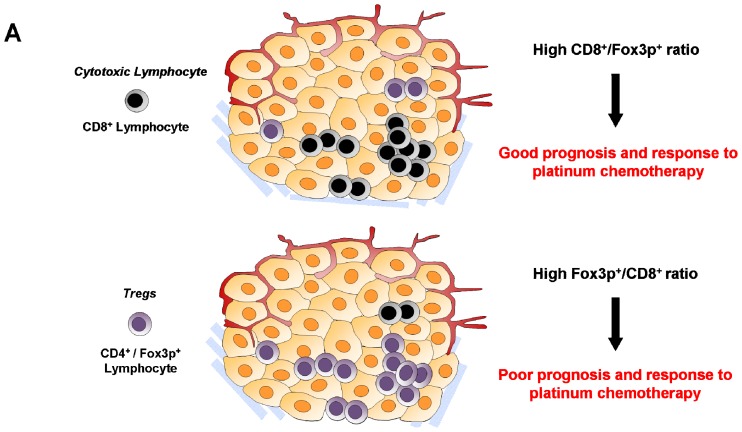

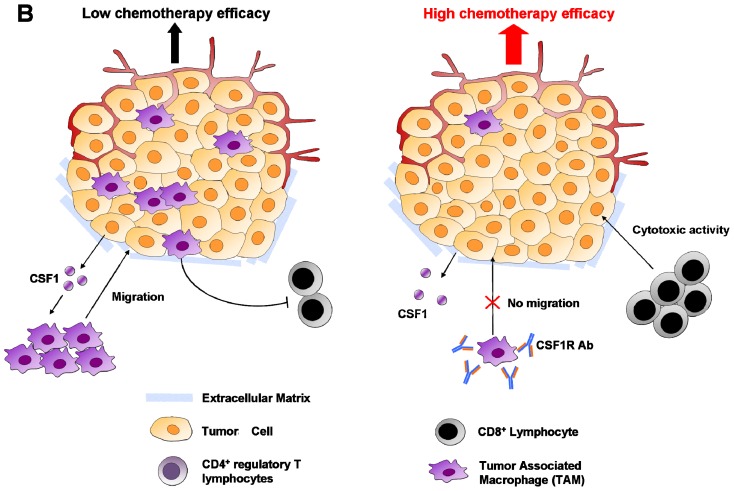
Effects of the stromal immune cell composition on the resistance to chemotherapy. (**A**) The sensitivity of non-small cell lung cancer to platinum chemotherapy is determined by the balance between regulatory CD4^+^/Fox3p^+^ lymphocytes and cytotoxic CD8^+^ lymphocytes; (**B**) Tumor associated macrophages (TAMs) are recruited into the tumor by secretion of colony stimulating factor 1 (CSF1) from tumor cells. TAMs inhibit the survival of CD8^+^ lymphocytes and lead to a reduced sensitivity to paclitaxel. Blocking CSF1 receptor (CSF1R) by a monoclonal antibody inhibits TAMs recruitment and allows a better cytotoxic activity and chemotherapy efficiency.

While TAMs are pro-tumoral and enhance chemoresistance, the presence of cytotoxic CD8^+^ lymphocytes (CTLs) is usually associated with a good prognosis and a better response to chemotherapy. Indeed, CTLs have an anti-tumor activity in secreting perforine or granzyme which induce apoptosis in tumoral cells. A high density of CD8^+^ cells has been correlated with a better response to chemotherapies in primary colorectal cancer or liver metastases [99,100]. Denkert *et al*. showed that the presence of lymphocytes infiltrates in breast cancer is correlated with an increased efficacy of anthracycline and taxane neoadjuvent chemotherapy [101] (Figure 1, Figure 3(A,B)).

Interactions between chemotherapy and CTLs are very tight and the efficacy of the latter can be increased after a first treatment. Indeed, a cyclophosphamide treatment in a mouse model of mesothelioma sensitizes tumor cells to the TRAIL-mediated death induced by CTLs [102]. Moreover, a chemotherapy treatment (based on paclitaxel, cisplatin or doxorubicin) leads to an up-regulation of mannose-6-phosphate receptors which increases the permeability of tumor cells to granzyme B released by CTLs [103]. Mattarollo *et al*. showed, in a mouse model of breast adenocarcinoma, that a doxorubicin treatment enhances the proliferation of IFN-γand IL-17 producing CD8^+^ T cells. In return, they demonstrated a positive correlation between the CD8α, CD8β and IFN-γ expressionb and the efficacy of doxorubicin treatment in patients with breast cancer [104]. Chemotherapy and CTLs thus present a synergical effect. The association between a classical chemotherapy treatment and immunotherapy present a very interesting way of therapeutic development.

### 6.3. γδ T Lymphocytes

γδ T lymphocytes are CD3^+^ cells expressing a TCR with γ and δ chains. They represent a small population of T lympochytes (5%) and show anti-tumor properties. Recently, the team of Zitvogel demonstrated that IL-17-producing γδ T lymphocytes were implied in the efficacy of chemotherapy. Indeed, in a mouse model lacking these γδ T cells, there is no IL-17 production and CTLs fail to invade tumor. The chemotherapy efficacy is thus impaired [105].

### 6.4. Regulatory T Cells (Treg)

Regulatory T cells, a Fox3p^+^ subpopulation of CD4^+^ T lymphocytes, are also implied in response to chemotherapy with controversial conclusions. Their presence is associated with a better survival exclusively in chemotherapy treated patients with an early breast cancer [106]. However, some other studies shown that the recruitment of Tregs in breast cancer can be associated with a bad clinical outcome and a higher risk of relapse [107,108]. In a recent work, Liu *et al*. demonstrated that the equilibrium between CTLs and Tregs in tumors is primordial to predict their response to chemotherapies. In an advanced non-small cell lung cancer context, a high Fox3p^+^/CD8^+^ ratio is associated with a poor response to platinum-based chemotherapy [106].

### 6.5. T Helpers (Th) Cells

CD4^+^ naïve T lymphocytes can, after the activation by antigen-presenting cells (APCs), differentiate into effector cells: T helpers (Th). The interactions with APCs and the nature of cytokine environment will define the subtype obtained: Th1, Th2 and Th17. These cells are implied in the activation of the immune system but differ in their cytokine production and biological functions.

Th1 cells are characterized by IFN-γ and TNF-α secretion and are responsible for the activation of CTLs or anti-tumoral macrophages. Tosilini *et al*. recently showed that the presence of Th1 cells was correlated with a prolonged disease-free survival in patients with colorectal cancer [109].

On the contrary, Th2 cells secrete IL-4 and enhance tumor progression in activating, for example, tumor-associated macrophages [110]. A few data directly links Th1 and Th2 cells with chemotherapy efficacy. However, the production of IL-4 (Th2 cytokine) is associated with resistance to chemotherapy and thyroid cancer [111].

Th17 secrete the pro-inflammatory cytokine IL-17 and present both anti-tumor and pro-tumor activities. The microenvironmental context is highly important in the interactions between Th17 cells and the tumor. However, many data link the IL-17 production, as previously cited for γδ lymphocytes, with the establishment of CTLs in the tumor bed and thus the anticancer efficacy [112].

As reviewed here and by Ding and Zhou, targeting CD4^+^ T cells constitutes a new way of immunotherapy in order to increase the chemotherapy efficacy [113].

### 6.6. Mast Cells

Among stromal cells of immune origin, mast cells (MCs) have been observed to infiltrate tumor masses. Indeed in lymphoid malignancies infiltrating MCs have been demonstrated to support clone survival and proliferation, and to negatively affect prognosis [114]. Mechanisms of tumor promotion involve the release of proangiogenic factor such as FGF-2 [115] or VEGF and matrix metalloproteinases (MMP9) but also of immunosuppressive cytokines like interleukin-10 [116]. As described before those molecules are also implicated in chemoresistance acquisition by tumor cells and MCs could be potential targets for chemotherapy treatment. They have been shown to be a prototypical off-target cell for imatinib therapy in cancer, due to their constitutively high expression of c-Kit, strong reliance on c-Kit signal for development and activation, and aforementioned tumor promoting activities [114].

## 7. Adipocytes

Although representing one of the most abundant parts of the breast cancer stroma, little is known about the cells that make up adipose tissue, adipocytes. What is certain is that they are active endocrine cells able to secrete growth factors and enhance tumor progression. Iyengar *et al*. showed that conditioned media from adipocytes could enhance the survival of breast cancer cells MCF-7 in serum starved media [117]. Results from a recent study supported the capacity of adipocytes to enhance chemoresistance in leukemia-bearing mice. In another study, 3L3-L1 adipocytes were shown to protect human leukemia cell lines from vincristine, nilotinib, daunorubin and dexamethasone, an effect which was independent of cell contact and associated with the increased expression of anti-apoptotic factors Pim-2 and Bcl-2 [118]. Bochet *et al*. demonstrated that cancer-associated adipocytes can also promote radioresistance and proposed that this effect could be due to a secretion of IL-6 and the phosphorylation of Chk1 [119].

**Figure 4:**
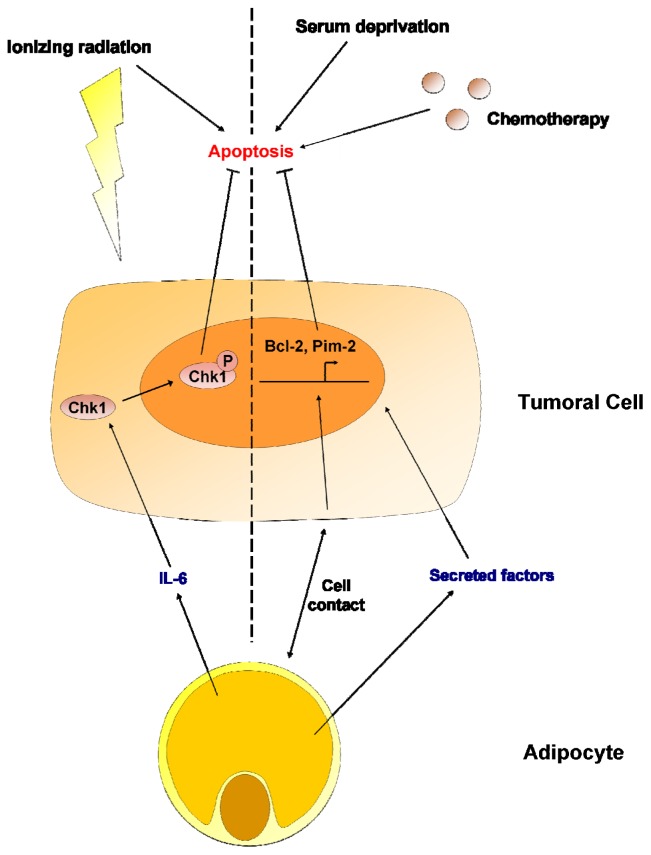
Effects of adipocytes on cancer cell resistance to apoptosis. The secretion of interleukin-6 (IL-6) by adipocytes activates Chk1 protein in breast cancer cells, which results in an enhanced resistance to radiotherapy. Adipocyte-secreted molecules enhance breast cancer cell resistance to serum deprivation via the activation of a pro-survival program. A cell-cell contact between adipocytes and leukemia cells is responsible for the transcription of anti-apoptotic proteins (Bcl-2 and Pim-2), which enhance the resistance to chemotherapy.

Considering the worldwide increase in the incidence of obesity, studying the interactions between adipocytes and cancer cells and their effects on chemotherapy and radiotherapy resistance is crucial in the discovery and development of new therapies (Figure 1,4).

## 8. miRNAs

More and more genes expression appears to be regulated by microRNAs (miRNAs). These small (approximately 22 bp) non-coding RNAs target specifically mRNA for cleavage, destabilization or inhibit their translation [120].

Expression of some miRNAs is deregulated in cancer and can be involved in chemoresistance through known or unknown mechanisms. MiR-Let-7e, miR-130a are among others associated with both cisplatin and taxol resistance in ovarian cancer [121]. Some miRNAs, like circulating miR-125b or miR-221 are found to be predictive markers of chemoresistance in breast cancer [122,123]. Other miRNAs are known to be implicated in chemoresistance by regulating genes involved in survival or cellular death. In mantle cell lymphoma cluster miR-17~92 allow reexpression of survival pathway PI3K/Akt by targeting negative regulators of this signaling pathway (protein phosphatase PHLPP2 and PTEN) [124]. This signaling pathway can also be activated via down-regulation of PPP2R1B (a subunit of protein phosphatase 2A) by miR-200c in esophageal cancers [125]. miR-155 is determinant in breast cancer chemoresistance by inhibiting specifically FOXO3a, which can induce cell death by up-regulation of apoptotic proteins, (BIM, p27, …) and repression of antiapoptotic molecule such as FLIP and Bcl-xL [126].

Moreover, miRNAs can be overexpressed in cancer cells leading to chemoresistance in response to microenvironment stimuli. Bourguignon and colleagues showed in head and neck squamous cell carcinoma (HNSCC) that miR-21 is up-regulated through the binding of hyaluronic acid to its receptor CD44 and promotion of Nanog-Stat 3 complex formation which allow transcriptional activation and expression of this miRNA. miR-21 up-regulation results in a decrease of a tumor suppressor protein (PDCD4), and an up-regulation of inhibitors of the apoptosis family of proteins (IAPs) as well as chemoresistance [127]. Stress conditions such as hypoxia lead to profound gene expression modifications and deregulations of several miRNAs [128]. For instance in HNSCC, miR-98 is up-regulated under hypoxia and leads to down-regulation of, at least, High mobility group A2 protein HMGA2 and potentiates chemoresistance to cisplatin and doxorubicin [129].

## 9. Conclusions

This review highlights the importance of simultaneously targeting both compartments of the tumor microenvironment (stromal and malignant cells) in cancer therapy. A number of targeted therapeutics is currently under development targeting not only cancer cells but also cells from the microenvironment such as fibroblasts, endothelial cells or mesenchymal stem cells. For example a tri-therapy currently under trial targets PDGFR (expressed by cancer cells, MSCs and fibroblasts), VEGFR (expressed by endothelial cells as well as cancer cells) and FGFR (expressed by fibroblasts and cancer cells). Two thirds of these novel targeted anticancer agents inhibit kinases, including EGFR, Src, and mammalian target of rapamycin (mTOR) activated in cancer cells as well as in microenvironment cells and now several V-ATPases inhibitors are under trial. Although these therapeutic approaches seem convincing they are associated with a lot of side effects associated with the elimination of cells not involved in cancer progression or in chemoresistance. Nevertheless, hope remains as many authors have described cells from the microenvironment which display several differences with their physiological parent cells. Indeed, when we compared some cancer stromal cells with the same cell from another organ and identified several genetic alterations. This enables distinguishing tumor associated fibroblasts (TAFs) from fibroblasts, carcinoma-associated MSCs from BM-MSCs (or ADSCs), and M1 macrophages from tumor-associated macrophages (TAMs). Differences can also be found between cells of the same origin but which have evolved differently depending on the organ and on the evolution of the associated pathologies e.g., Hospicells/CA-MSCs, myofibroblasts/TAFs. This highlights the broad versatility and plasticity of microenvironment cells. Parental cells (such as macrophages, neutrophils or MSCs) can mostly be found with anti tumoral properties. They can however polarize into different states depending on the tumor and acquire pro-tumoral functions when they may also influence chemoresistance acquisition and act on the apoptosis pathways of tumor cells. However, what actually causes this switch of a microenvironment cell from being tumor inhibiting to tumor-promoting remains a mystery. It is feasible that microenvironment cells (such as MSCs) could be activated during chemotherapy treatment (platinium analog) and secrete factors that protect tumor cells against a range of chemotherapeutics. The future goal will be to characterize phenotypic differences between cancer-activated stromal cells and their physiological counterparts in order to define therapies which will specifically target activated stromal cells. Studying the impact of defined genetic alterations on therapeutic response in native tumor microenvironments will be critical for effective drug development, personalized cancer regimens, and the rational design of combination therapies.

Although the manner in which stroma cells activate cancer cells to promote cancer progression and chemoressistance are described, little is known about the mechanisms by which stromal cells are first activated to induce these effects. There is a cross-talk between cancer cells and stromal cells but factors produced by the stromal cells and the cancer cells vary depending on the context and on the microenvironment compartment. As an example, Roodhardt *et al*. described an induction of cancer cell chemoresistance by the MSCs through the release of a fatty acid while Castells *et al*. propose that chemoresistance could be acquired by cancer cells through release of IL-6 and IL-8 by macrophages which were activated by an unidentified secreted factor produced by MSCs in human ovarian cancers [4]. Therefore, given the mounting evidence supporting the diversity of mechanisms by which microenvironment cells enhance tumor chemoresistance, inhibiting the factors released by stroma cells in a clinical setting will be challenging.

Finally, the implication of fatty acids in cell signaling and cross-talk between cancer cells and their microenvironment and thus their importance in chemotherapy resistance has been demonstrated in several recent studies. Their potential as key modulators of treatment response warrants further fundamental and translational investigation, which could result in them becoming a promising target for therapy.

The mechanisms behind chemosensitivity and chemoresistance are being unraveled however managing patients who have primary chemorefractory disease or relapse after the most aggressive salvage therapies remains a challenge. Conventional anticancer drug screening is typically performed in the absence of accessory cells of the tumor microenvironment, which can profoundly alter antitumor drug activity. Preclinical drug testing in the absence of relevant tumor microenvironment interactions may overestimate potential clinical activity, thus explaining at least in part the gap between preclinical and clinical efficacy in cancers [130,131].

The screening of candidate anticancer agents to enrich preclinical pipelines with potential therapeutics able to overcome stroma-mediated drug resistance and act in a synthetically lethal manner must be performed in the context of tumor-stroma interactions.
